# Treg and neutrophil extracellular trap interaction contributes to the development of immunosuppression in sepsis

**DOI:** 10.1172/jci.insight.180132

**Published:** 2024-06-18

**Authors:** Yuxin Shi, Dan Wu, Yanghanzhao Wang, Yuwen Shao, Fu Zeng, Di Zhou, Hao Zhang, Changhong Miao

**Affiliations:** 1 Department of Anesthesiology, Zhongshan Hospital, Fudan University, Shanghai, China.; 2 Shanghai Key Laboratory of Perioperative Stress and Protection, Shanghai, China.; 3 Department of Anesthesiology, Shanghai Medical College, Fudan University, Shanghai, China.

**Keywords:** Immunology, Inflammation, Adaptive immunity, Immunotherapy, T cell development

## Abstract

The excessive formation and release of neutrophil extracellular traps (NETs) in sepsis may represent a substantial mechanism contributing to multiorgan damage, which is associated with a poor prognosis. However, the precise role of NETs in mediating the transition from innate immunity to adaptive immunity during the progression of inflammation and sepsis remains incompletely elucidated. In this study, we provide evidence that, despite a reduction in the number of CD4^+^ T cells in the late stage of sepsis, there is a notable upregulation in the proportion of Tregs. Mechanistically, we have identified that NETs can induce metabolic reprogramming of naive CD4^+^ T cells through the Akt/mTOR/SREBP2 pathway, resulting in enhanced cholesterol metabolism, thereby promoting their conversion into Tregs and augmenting their functional capacity. Collectively, our findings highlight the potential therapeutic strategy of targeting intracellular cholesterol normalization for the management of immunosuppressed patients with sepsis.

## Abstract 2

Neutrophil extracellular traps drive the differentiation and function of Tregs through enhanced AKT/mTOR/SREBP2 signaling and promotion of cholesterol acquisition pathways.

## Introduction

Sepsis stands as a leading cause of mortality among patients admitted to intensive care units (ICUs) (1). The updated definition of sepsis underscores a dysregulated host response to infection, characterized by a concomitant imbalance of excessive inflammation and immune suppression, which serves as the primary driver of multiorgan dysfunction and mortality (2). Recent research has demonstrated the role of immune suppression, stemming from excessive lymphocyte apoptosis, as a pivotal factor in sepsis pathogenesis and prognosis (3). This immunosuppression heightens the risk of secondary nosocomial infections among patients with sepsis (4). Tregs, a distinct subset of immune cells, exert their primary function by suppressing immune responses, maintaining immune homeostasis, and facilitating self-tolerance (5). Evidence suggests that during the early stages of sepsis, there is a reduction in Treg numbers, leading to heightened immune responses and exacerbation of inflammation. Conversely, in the late stages of sepsis, there is a marked increase in Treg numbers, presumably as a regulatory response to excessive inflammation (6, 7). However, a comprehensive mechanistic understanding of Tregs’ role in sepsis development remains elusive.

Neutrophils, pivotal constituents of the innate immune system, contribute to the excessive inflammation in sepsis by releasing proteases and ROS (8, 9). Upon stimulation by external pathogens, neutrophils form neutrophil extracellular traps (NETs), serving as a crucial immune barrier against invading pathogens (9). However, excessive NETosis can have detrimental effects on sepsis despite its efficacy in trapping and killing bacteria (10–12). Previous studies have demonstrated increased NET generation in the early stage of sepsis, persisting throughout the disease progression (13, 14). Despite the substantial role of NETs in immune and inflammatory regulation, their precise contribution and regulatory mechanisms in late sepsis-induced immunosuppression remain unclear.

In sepsis, immunosuppression can affect the function of Tregs by influencing immune cell metabolism (15). Tregs can generate the necessary cellular building blocks through anabolic glycolysis to promote cell proliferation and produce adenosine triphosphate energy through oxidative phosphorylation (OXPHOS) driven by mitochondrial catabolism fatty acid oxidation to support activation and inhibition functions (15). Within Tregs, the master transcription factor forkhead box protein P3 (Foxp3) inhibits the expression of Myc, thereby inhibiting glycolysis and promoting OXPHOS (16). However, the metabolic state and dynamic changes of Tregs in the process of sepsis remain unclear and need further investigation. Thus, the metabolic pathways that regulate Treg development have emerged as potential immunotherapeutic targets for sepsis.

The immune processes related to Tregs are intricately complex and interrelated in sepsis. So far, whether and how NETs affect Tregs’ metabolic mechanisms in sepsis remains poorly understood. Our study reveals an elevation of NETs in a late-stage mouse model of sepsis, contributing to increased Treg differentiation, a process associated with altered cholesterol metabolism in naive CD4^+^ T cells mediated by NETs.

## Results

### Enhanced NET formation contributes to multiple organ dysfunction syndrome in sepsis.

**Figure 1:**
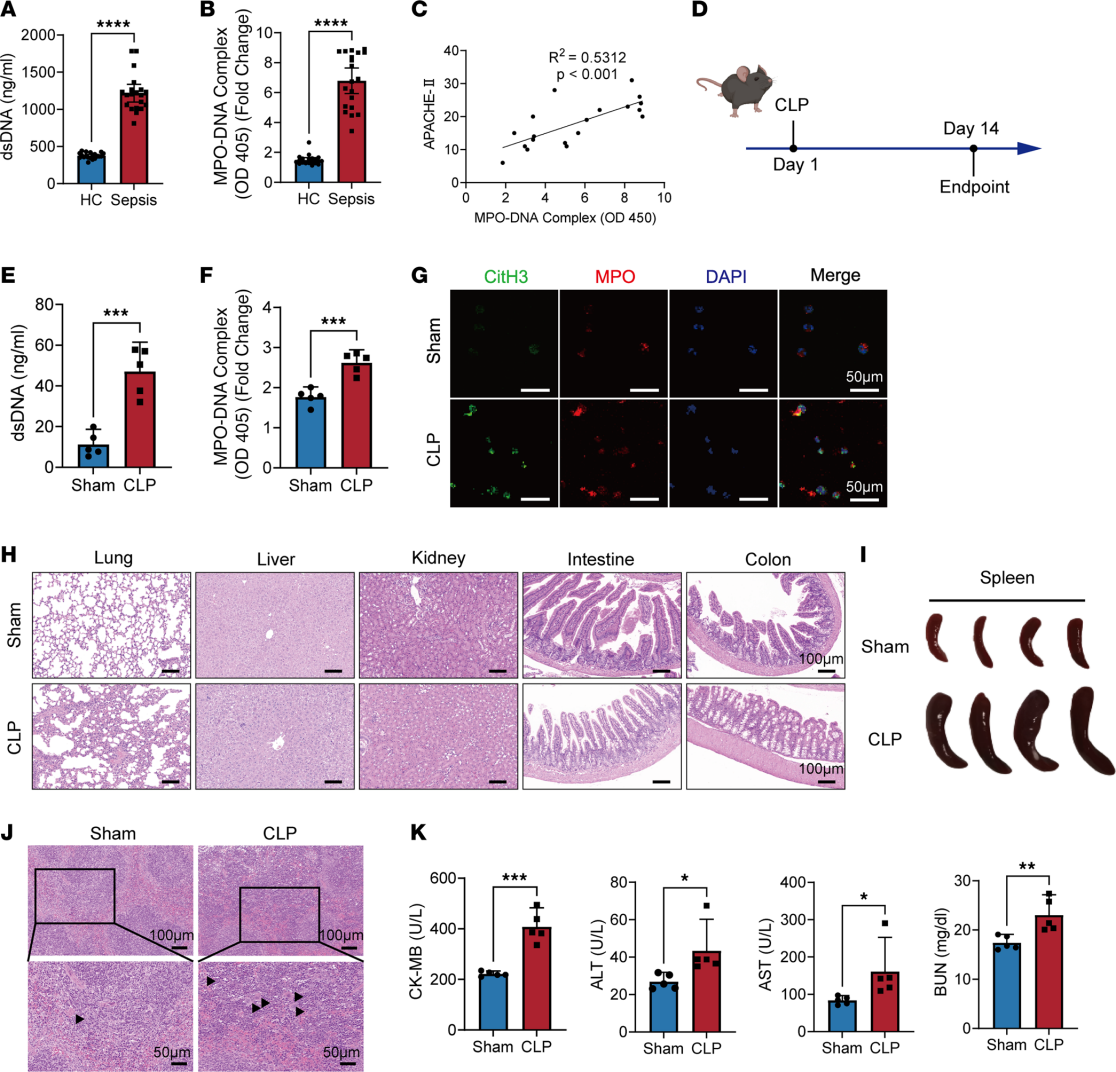
NET formation is enhanced during sepsis, leading to multiple organ dysfunction syndrome. (**A**) dsDNA levels and (**B**) MPO-DNA complex in sera of patients with sepsis (*n* = 20) and individuals acting as healthy controls (HC) (*n* = 20). (**C**) Correlation curve between the MPO complex and APACHE-II scores (*n* = 19, *r*^2^ = 0.5312, *P* < 0.001). (**D**) Experimental design of mouse postsepsis immunosuppression (PICS) model in the immunosuppression period. (**E**) dsDNA levels and (**F**) MPO-DNA complex in plasma of CLP mice (*n* = 5). (**G**) Representative immunofluorescence images of NETs released by bone marrow neutrophils isolated from mice treated with CLP and mice in the sham group. Scale bars: 50 μm. (**H**) Representative H&E staining of lungs, livers, kidneys, small intestines, and colons in septic and healthy mice. Scale bars: 100 μm. (**I**) Representative image of spleens from CLP mice and the sham group. (**J**) Representative H&E staining of spleens from septic and healthy mice. Scale bars: 100 μm (top); 50 μm (bottom). (**K**) Levels of organ injury markers CK-MB, BUN, ALT and AST in plasma of mice models (*n* = 5). Each bar represents mean ± 95% CI. Data comparison between 2 groups was analyzed by unpaired *t* test. *P < 0.05, **P < 0.01, ***P < 0.001, ****P < 0.0001.

First, NETs were confirmed to be involved in the sepsis process. Levels of dsDNA and myeloperoxidase (MPO) complexes were prominently elevated in patients with sepsis compared with those in individuals acting as healthy controls (Figure 1, A and B). Moreover, MPO-DNA levels positively correlated with APACHE-II scores in patients with sepsis (Figure 1C). To delve deeper, a late-stage sepsis mouse model was established via cecal ligation and puncture (CLP) (Figure 1D). Similarly, compared with the sham group, plasma levels of dsDNA and MPO were higher in CLP mice (Figure 1, E and F). Neutrophils isolated from septic mice exhibited an increased capacity for NET formation, as evidenced by coexpression of CitH3 and MPO upon stimulation with PMA (Figure 1G). After 2 weeks, CLP mice exhibited more severe organ injuries, with extensive lymphocytic infiltration in multiple organs (Figure 1H). Additionally, CLP mice presented with notably swollen spleens (Figure 1I), characterized by the presence of multinucleated giant cells (Figure 1J), alongside distinctly elevated plasma levels of organ injury markers, including cardiac creatine kinase muscle and brain isoenzyme (CK-MB), renal blood urea nitrogen (BUN), hepatic alanine transaminase (ALT), and aspartate transaminase (Figure 1K). These findings collectively suggest that enhanced NET formation contributes to multiple organ dysfunction in sepsis.

### Increased Treg numbers during the immunosuppressive phase of sepsis.

**Figure 2:**
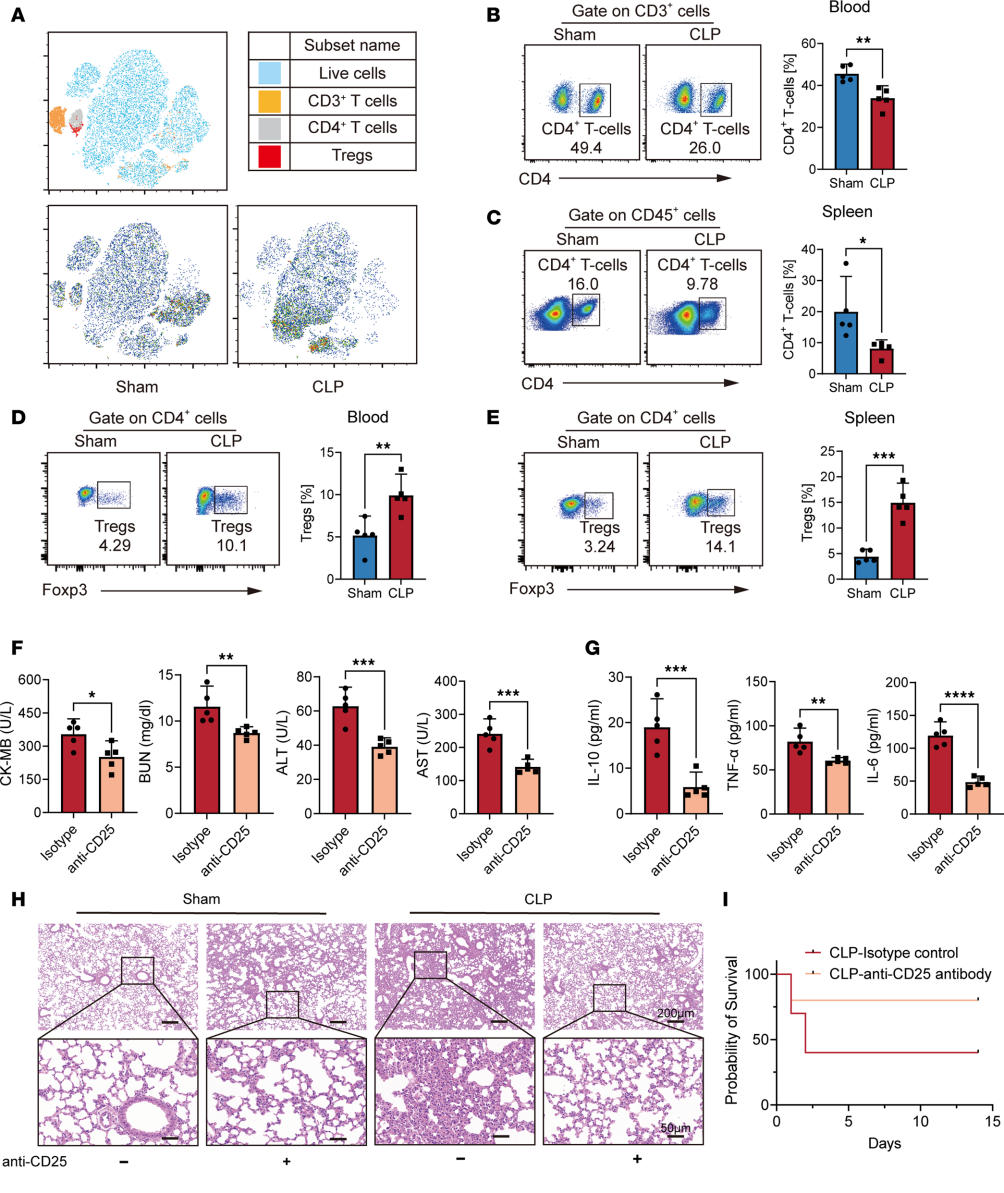
Treg numbers increase during sepsis immunosuppressive phase. (**A**) Composition of key immune cells in T subset in CLP spleens (age = 12 weeks, *n* = 5). (**B**) The percentage of CD4^+^ T cells in blood was determined on day 14 after CLP (*n* = 5). (**C**) The percentage of CD4^+^ T cells in spleen was determined on day 14 after CLP (*n* = 5). (**D**) The percentage of Tregs in blood was determined on day 14 after CLP (*n* = 5). (**E**) The percentage of Tregs in spleen was determined on day 14 after CLP (*n* = 5). (**F**) The plasma levels of organ injury markers and (**G**) cytokines TNF-α, IL-6, and IL-10 in CLP mice with or without anti-CD25 antibody injection (age = 12 weeks, *n* = 5). (**H**) Representative images of H&E staining of lung tissue sections from the sham group and CLP mice with or without anti-CD25 antibody treatment. Scale bars: 200 μm (top); 50 μm (bottom). (**I**) Survival analysis of septic mice using anti-CD25 antibody (*n* = 10, *P* < 0.05). The survival analysis was determined using the Kaplan-Meier method and the log-rank test. Each bar represents mean ± 95% CI. Data comparison between 2 groups was analyzed by unpaired *t* test. *P < 0.05, ** *P* < 0.01, ***P < 0.001, **** *P* < 0.0001.

We initially investigated the immune landscape within the spleen T cell microenvironment using a murine model of late sepsis. Specifically, we assessed key immune cell populations in the septic spleen on day 14, indicative of immunosuppressive events (Figure 2A and Supplemental Figure 1A; supplemental material available online with this article; https://doi.org/10.1172/jci.insight.180132DS1). Consistent with previous studies, we observed an evident reduction in CD4^+^ T cell numbers in both the blood and spleens of CLP-treated mice compared with those in the sham group (Figure 2, B and C). Subsequently, we quantified changes in the proportion of the major CD4^+^ T cell subsets in septic mice. Notably, despite the overall decrease in total CD4^+^ T cell count, the proportion of Tregs exhibited a remarkable increase (Figure 2, D and E).

To further explore the role of Tregs in sepsis progression, we depleted Tregs using anti-CD25 antibody treatment in the CLP mouse model (Supplemental Figure 1B). This intervention resulted in reduced levels of biochemical organ injury markers (Figure 2F) and inflammatory cytokines (IL-6, IL-10, and TNF-α), indicating attenuation of the sepsis-induced inflammatory response (Figure 2G). Additionally, we observed diminished leukocyte infiltration in lung sections of CLP mice after 14 days (Figure 2H). Furthermore, CLP mice treated with anti-CD25 antibodies exhibited prolonged survival compared with that in the control group (Figure 2I). Collectively, these findings suggest that elevated Treg levels observed in late-stage sepsis play an important role in immunosuppression and disease progression.

### NETs regulate Treg differentiation and function in sepsis.

**Figure 3:**
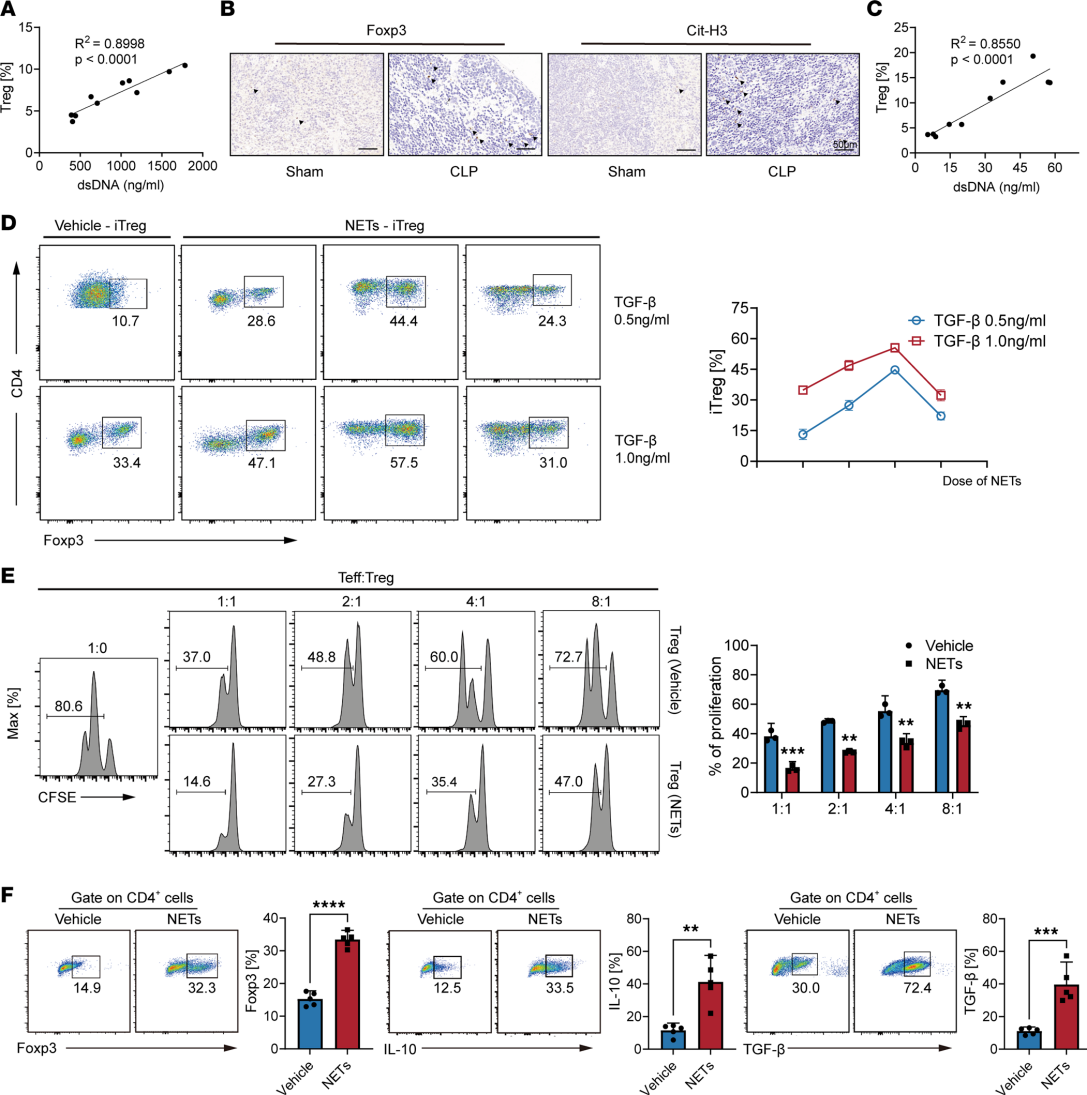
A positive correlation between NETs and Tregs in sepsis and NETs promote Treg differentiation in vitro. (**A**) Correlation between plasma dsDNA and proportion of Tregs in healthy controls and patients with sepsis (*n* = 5, *r*^2^ = 0.8998, *P* < 0.0001). (**B**) Representative images of immunohistochemical staining of the spleens from mice treated with CLP (black arrowheads indicate Foxp3^+^ cells). Scale bars: 50 μm. (**C**) Correlation between plasma dsDNA and the proportion of Tregs in septic mice (age = 12 weeks, *n* = 10, *r*^2^ = 0.8550, *P* < 0.0001). (**D**) Differentiation of iTregs treated with NETs or vehicle in vitro (*n* = 3). (**E**) In vitro suppressive assay of Tregs pretreated with NETs or vehicle (*n* = 3). (**F**) Representative flow cytometric analysis of Foxp3, IL-10, and TGF-β percentages in naive CD4^+^ T cells (*n* = 5). Each bar represents mean ± 95% CI. Data comparison between 2 groups was analyzed by unpaired *t* test. **P < 0.01, ***P < 0.001, **** *P* < 0.0001.

Given the evident increase in NETs during late-stage sepsis, we explored the potential relationship between NETs and elevated Treg levels in late sepsis. We first found a positive correlation between serum levels of dsDNA and Tregs in patients with sepsis (Figure 3A). Immunohistochemical analysis revealed a higher presence of Cit-H3 (a marker for NETs) and increased Foxp3 levels in septic mouse spleens (Figure 3B). Indeed, a positive correlation was observed between plasma levels of dsDNA and Foxp3 in septic mice, further supporting the association between NETs and Tregs in late sepsis (Figure 3C). Thus, we concluded that NETs may contribute to the observed high Treg levels during late-stage sepsis.

In vitro studies demonstrated that Tregs can be differentiated from naive CD4^+^ T cells under T cell receptor (TCR) activation in the presence of TGF-β and IL-2 (17). Previous research has shown that NETs can influence Treg differentiation (18). To investigate this further, we isolated naive CD4^+^ T cells and exposed them to increasing concentrations of NETs and TGF-β to cause induced Treg (iTreg) differentiation. A noticeable increase in iTreg differentiation with higher doses of NETs was observed compared with that of the control group (Figure 3D). When the concentration of NETs reached its maximum, the proportion of iTregs decreased, leading us to speculate that high concentrations of NETs may cause the disruption of CD4^+^ T cell regulatory function, thereby affecting their differentiation and activity. Additionally, we examined the effect of NETs on the function of iTregs by conducting in vitro suppression assays on isolated Tregs pretreated with NETs or vehicle for 48 hours. Notably, NET-treated Tregs exhibited enhanced suppressive function (Figure 3E). Furthermore, NET-induced Tregs demonstrated upregulation of Foxp3, IL-10, and TGF-β (Figure 3F). These findings collectively demonstrate that NETs influence the differentiation and function of Tregs within the sepsis microenvironment.

### NETs enhance cholesterol metabolism in Tregs through SREBF2.

**Figure 4:**
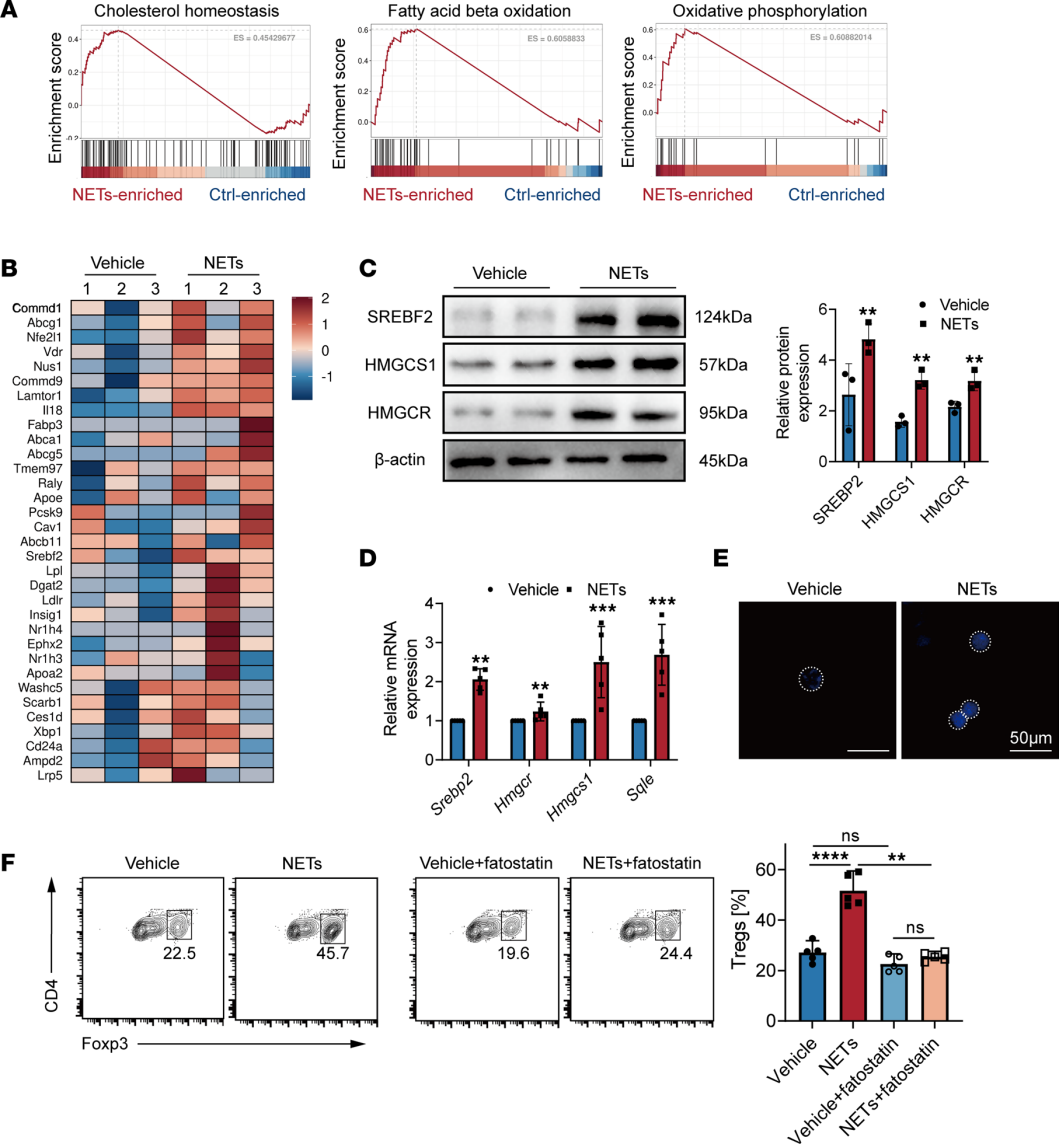
NETs enhance cholesterol metabolism in Tregs through the SREBP2 pathway. (**A**) Top enriched pathways in NET-treated naive CD4^+^ T cells, compared with those in the nontreated group. (**B**) Heatmap of naive CD4^+^ T cells with or without NET treatment in cholesterol homeostasis. (**C**) Western blot images of SREBP2-regulated gene expression in naive CD4^+^ T cells. (**D**) The relative gene expression of SREBP2-regulated gene expression in naive CD4^+^ T cells. (**E**) Filipin staining of naive CD4^+^ T cells treated with or without NETs. Scale bars: 50 μm. (**F**) iTreg differentiation in NET-treated or control group with or without fatostatin treatment (*n* = 5). Each bar represents mean ± 95% CI. Data comparison between 2 groups was analyzed by unpaired *t* test. Statistical analysis for 3 or more groups was preformed using 1-way ANOVA. *P < 0.05, **P < 0.01, ***P < 0.001, ****P < 0.0001.

Apart from the capacity of NETs to influence the differentiation and function of Tregs in the late stage of sepsis, we also elucidated the specific mechanisms underlying the differentiation of naive CD4^+^ T cells into Tregs in the presence of NETs. Gene set enrichment analysis was conducted between naive CD4^+^ T cells induced with NETs or PBS for 72 hours. Pathway analysis revealed that cholesterol homeostasis, fatty acid β-oxidation, and OXPHOS were the top enriched signatures in NET-induced Tregs (Figure 4A). Metabolic reprogramming of T cells following activation is considered an integral component of Treg function and polarization. Resting naive or memory T cells, which primarily rely on OXPHOS and fatty acid oxidation for energy generation, undergo a rapid transition toward biosynthetic metabolic pathways, such as glycolysis and cholesterol biosynthesis, upon activation (19, 20). Studies demonstrate that enhanced cholesterol metabolism can facilitate T cell differentiation (21). The SREBPs are transcription factors that play a crucial role in regulating cholesterol and lipid metabolism (22). The SREBP cleavage-activating protein (SCAP) is involved in the regulation of SREBPs (23). Our study suggests that the SCAP/SREBP pathway may influence Treg function, potentially through the regulation of cholesterol-related genes, as indicated by heatmap analysis (Figure 4B). Considering the importance of SREBP2 in Treg differentiation and function, the expression of *Srebp2* and other genes involved in cholesterol metabolism was examined, revealing an obvious increase in the mRNA and protein levels of these genes after NET treatment (Figure 4, C and D).

Our findings suggest a link between lipid biosynthetic pathways, particularly cholesterol, under NET treatment; therefore, further biochemical assessments were performed to confirm the presence of cholesterol in NET-treated cells. Confocal microscopy analysis using filipin staining on day 3 of differentiation revealed a noticeable increase in intracellular cholesterol levels (Figure 4E). To mechanistically validate the role of SREBP2 in Treg development under NET treatment, we cotreated naive CD4^+^ T cells with a SREBP reductase inhibitor (fatostatin), which binds to SCAP and inhibits the ER-Golgi translocation of SREBPs. While Foxp3 expression was induced by NETs, SREBP2 inhibition blocked its activation (Figure 4F). Collectively, our data indicate that NETs regulate cholesterol metabolism in naive CD4^+^ T cells through SREBP2, thereby promoting their differentiation into Tregs.

### TLR4 and mTOR pathway are required in NET-related cholesterol reprogramming in naive CD4^+^ T cells.

**Figure 5:**
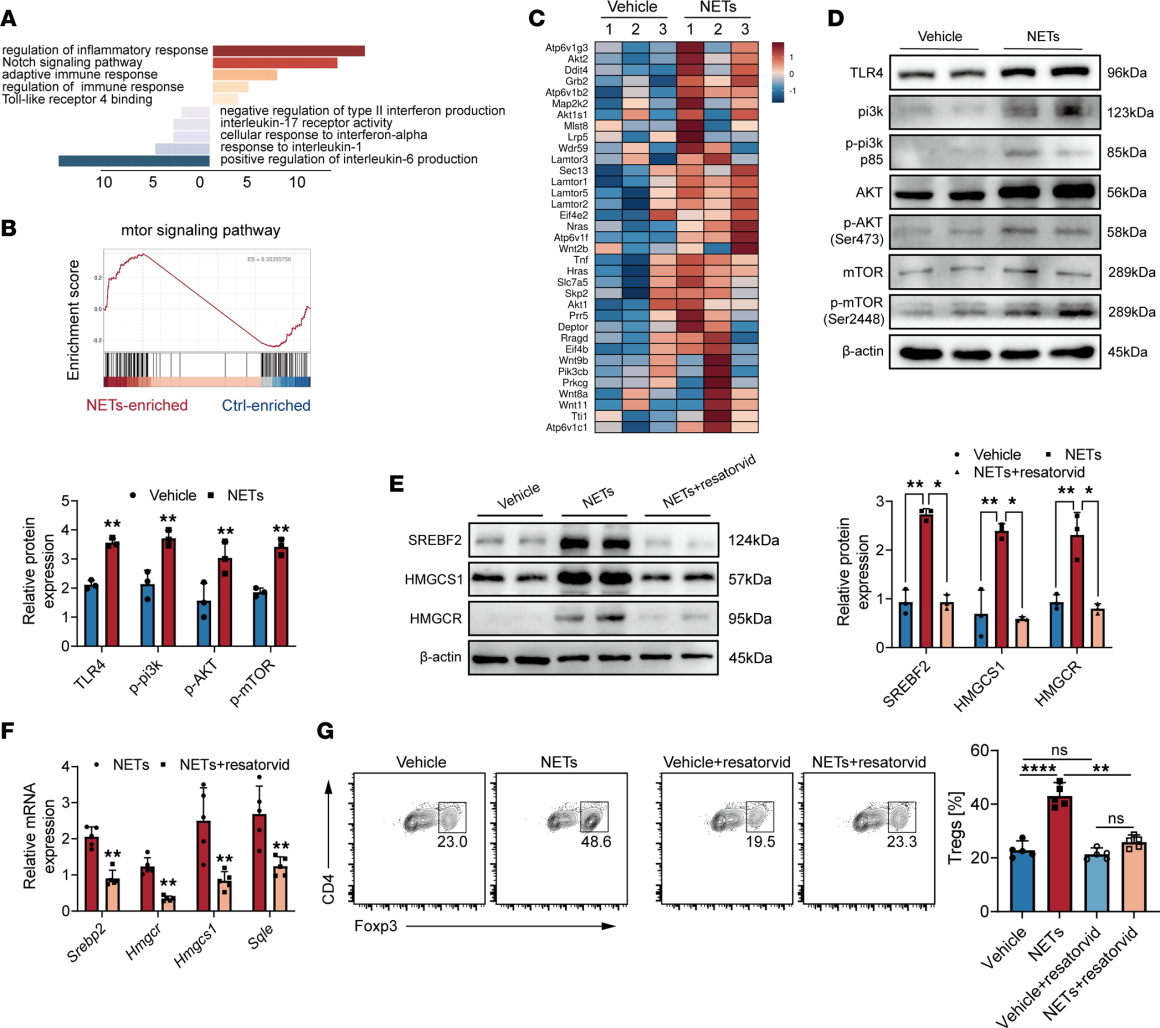
TLR4 and the mTOR pathway are required in NET-related cholesterol reprogramming in naive CD4^+^ T cells. (**A**) Top receptor and cytokine pathway enrichment analysis in naive CD4^+^ T cells with or without NET treatment. (**B** and **C**) Gene set enrichment analysis and heatmap of signature genes related in the mTOR signaling pathway. (**D**) Western blot images of TLR4/PI3K/Akt/mTOR pathway expression in naive CD4^+^ T cells. (**E**) Western blot images of SREBP2-regulated gene expression in naive CD4^+^ T cells. (**F**) The relative gene expression of SREBP2-regulated gene expression in naive CD4^+^ T cells was tested by qPCR. (**G**) iTreg differentiation assessed in NETs or vehicle-treated naive CD4^+^ T cells with or without resatorvid treatment (*n* = 5). Each bar represents mean ± 95% CI. Data comparison between 2 groups was analyzed by unpaired *t* test. Statistical analysis for 3 or more groups was preformed using 1-way ANOVA. *P < 0.05, **P < 0.01, ***P < 0.001, ****P < 0.0001.

To gain mechanistic insights into NET-induced differentiation of naive CD4^+^ T cells, we conducted a differential analysis. The top noticeably altered receptor and cytokine-related pathways were identified, along with the upregulation of TLR4 binding, consistent with previous studies (Figure 5A) (18). TLR4 activation, typically triggered by LPS, can stimulate mTOR signaling, which may occur through various pathways, including the PI3K/Akt/mTOR axis (24, 25). The downstream effects of TLR4-induced mTOR activation are mainly in areas of immune cell function, proliferation, and cytokine production (26). Importantly, the mTOR pathway has been implicated in Treg development and function (26), with TLR4 activation indirectly influencing Treg function through mTOR modulation (27). Moreover, pathway and heatmap analysis revealed enrichment of the mTOR signaling pathway in NET-treated naive CD4^+^ T cells (Figure 5, B and C). NET treatment resulted in increased TLR4, p-PI3K, p-mTOR, and p-AKT expression (Figure 5D). Collectively, these results indicate that NETs modulate Treg differentiation via the mTOR pathway.

To mechanistically validate the role of cholesterol in Treg development in the context of NET stimulation, we employed resatorvid, an inhibitor of the TLR4 signaling pathway. Resatorvid downregulates MyD88 and TRIF signaling molecules, which are downstream of TLR4. In our study, treatment of naive CD4^+^ T cells with NETs and resatorvid prominently inhibited the SREBP2 pathway at both the protein and mRNA levels (Figure 5, E and F). Furthermore, the differentiation of NET-induced Tregs was also reduced in vitro upon resatorvid treatment (Figure 5G). These findings collectively support the conclusion that NETs influence SREBP2-regulated cholesterol metabolism through the TLR4 pathway, thereby affecting Treg differentiation.

### NET degradation reduces Treg activity and organ dysfunction during sepsis.

**Figure 6:**
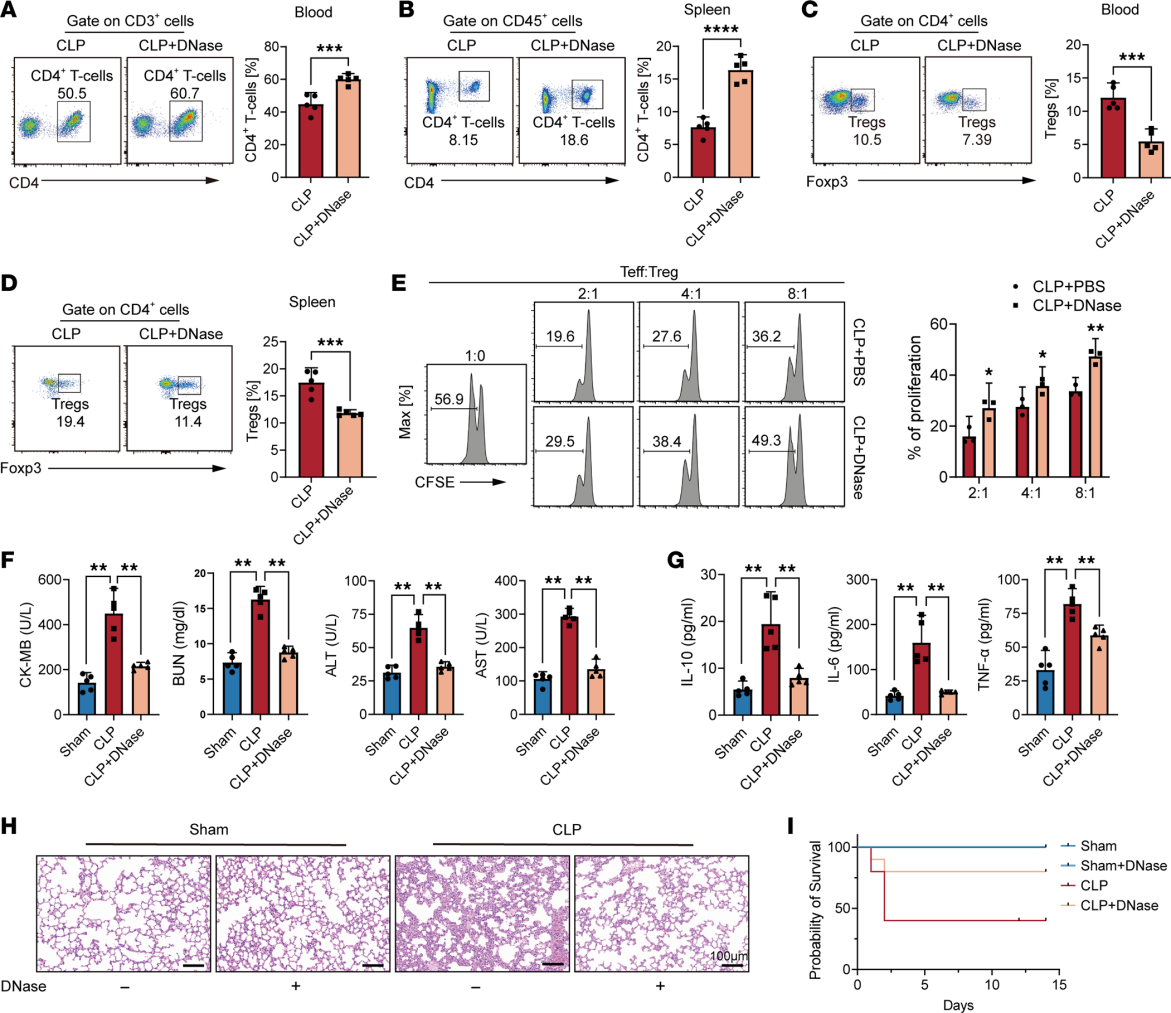
NET degradation reduces Treg activity and organ dysfunction during sepsis. (**A**) The proportion of total CD4^+^ T cells in blood with or without DNase treatment on day 14 after sepsis (*n* = 5). (**B**) The proportion of CD4^+^ T cells in spleen on day 14 after sepsis (*n* = 5). (**C**) The proportion of Tregs in blood on day 14 after sepsis (*n* = 5). (**D**) The proportion of Tregs in spleen on day 14 after sepsis (*n* = 5). (**E**) In vitro suppressive assay of Tregs isolated from CLP and CLP+DNase mice (*n* = 3). (**F**) The murine plasma levels of blood chemical indicators and (**G**) cytokines IL-10, IL-6, and TNF-α (*n* = 5). (**H**) Representative images of H&E staining of mouse lung tissue sections. Scale bars: 100 μm. (**I**) Survival analysis of septic mice treated with or without DNase (*n* = 10, *P* < 0.05). The survival analysis was determined using the Kaplan-Meier method and the log-rank test. Each bar represents mean ± 95% CI. Data comparison between 2 groups was analyzed by unpaired *t* test. Statistical analysis for 3 or more groups was preformed using 1-way ANOVA. *P < 0.05, **P < 0.01, ***P < 0.001.

To validate the contribution of NETs to the population and function of Tregs in sepsis in vivo, mice were treated with DNase to degrade NETs (Supplemental Figure 1C). Herein, the proportion of total CD4^+^ T cells increased in both the blood and spleen following NET degradation by DNase injection (Figure 6, A and B). Furthermore, CD4^+^Foxp3^+^ Treg levels were reduced in septic mice treated with DNase (Figure 6, C and D). To investigate the effect of NETs on Treg function in vivo, we isolated Tregs from DNase-treated CLP mice. These Tregs exhibited weakened suppressive function against effector T cells (Teffs) compared with the CLP groups (Figure 6E).

A positive correlation exists between the concentration of NETs and multiorgan dysfunction during sepsis (12). In our study, mice treated with DNase exhibited lower plasma levels of organ injury markers, including cardiac CK-MB, hepatic ALT and AST, and renal BUN, compared with septic mice (Figure 6F). Furthermore, the persistent inflammatory response, as indicated by low plasma concentrations of TNF-α, IL-6, and IL-10, was alleviated in the absence of NETs, which were substantially elevated in septic mice (Figure 6G), whereas administration of DNase mitigated lung injury (Figure 6H). Importantly, septic mice treated with DNase demonstrated an improved survival rate compared with that of mice with CLP-induced sepsis (Figure 6I). These findings strongly suggest that NETs contribute to septic immunosuppression, and therapies targeting NETs hold promise as protective interventions.

### Inhibition of SREBP2 prevents organ dysfunction in sepsis.

**Figure 7:**
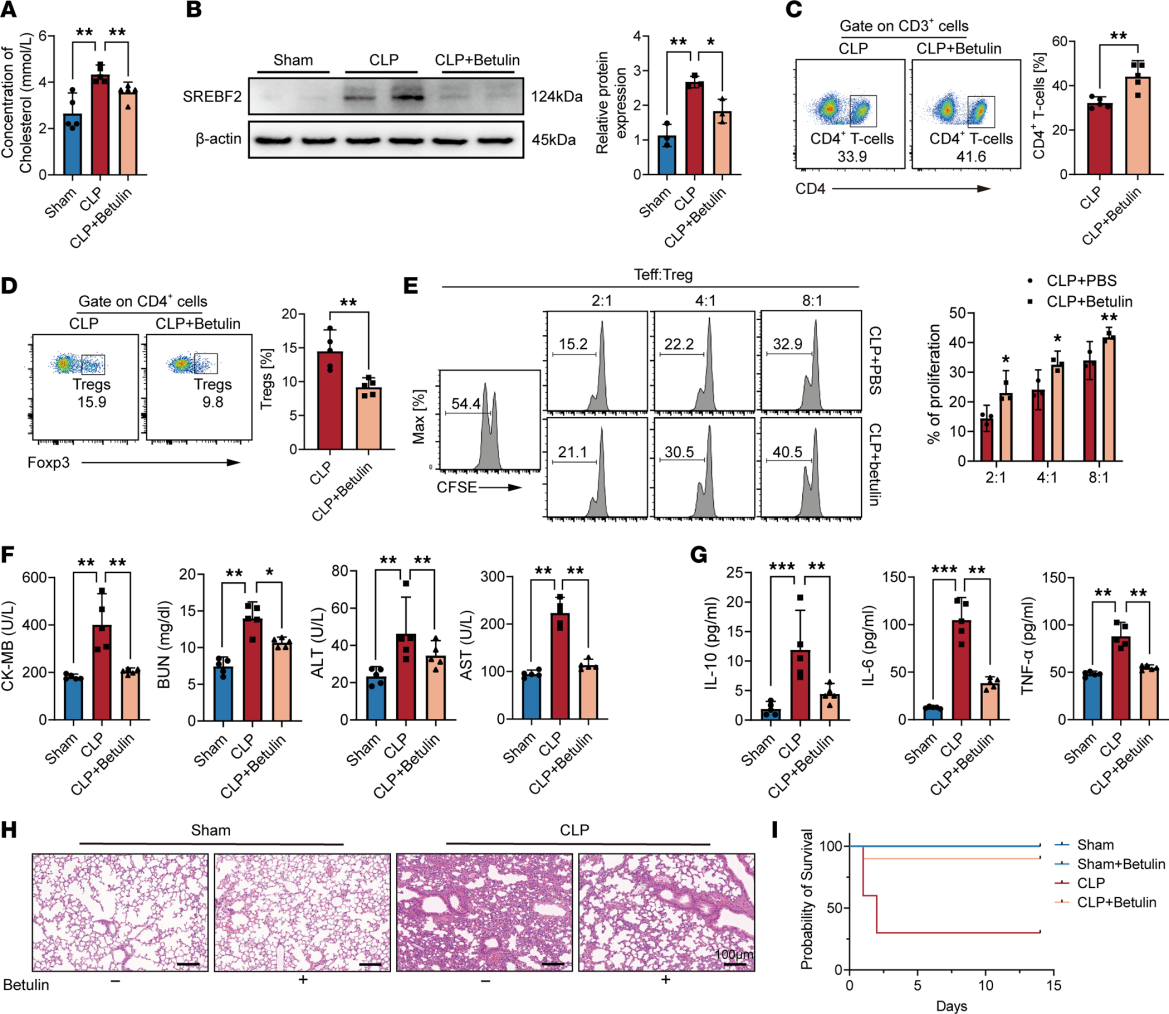
Inhibition of SREBP2 prevents organ dysfunction in sepsis. (**A**) Concentration of cholesterol in sera from the sham group and CLP mice treated with or without betulin (*n* = 5). (**B**) Western blot images of SREBF2 expression in CD4^+^ T cells. (**C**) Percentage of CD4^+^ T cells in blood of CLP mice with or without betulin treatment on day 14 after CLP (*n* = 5). (**D**) Flow cytometric analysis of Foxp3 percentage in CD4^+^ T cells on day 14 after CLP (*n* = 5). (**E**) In vitro suppressive assay of Tregs isolated from CLP mice treated with betulin or PBS (*n* = 3). (**F**) The circulating levels of organ injury markers and (**G**) cytokines from murine plasma (*n* = 5). (**H**) Normal and septic lung sections with or without betulin treatment were subjected to H&E staining. Scale bars: 100 μm. (**I**) Mice pretreated with betulin or PBS induced by CLP, and were then followed for survival analysis (*n* = 10). The survival analysis was determined using the Kaplan-Meier method and the log-rank test. Each bar represents mean ± 95% CI. Data comparison between 2 groups was analyzed by unpaired *t* test. Statistical analysis for 3 or more groups was preformed using 1-way ANOVA. *P < 0.05, **P < 0.01, ***P < 0.001.

Betulin has been demonstrated to effectively inhibit the maturation of SREBP by inducing SCAP and Insig, leading to a reduction in cholesterol and fatty acid biosynthesis (28). Therefore, we conducted further investigations to explore whether pharmacological inhibition of SREBP2 by betulin could prevent organ dysfunction in the context of sepsis (Supplemental Figure 1D). Initially, we examined total cholesterol concentration in mouse sera (Figure 7A) and isolated CD4^+^ T cells from the spleens of mice. Subsequent Western blot analysis confirmed the inhibition of the SREBP2 pathway in CLP mice treated with betulin (Figure 7B and Supplemental Figure 1E). Remarkably, we observed an increased proportion of CD4^+^ T cells in the peripheral blood of septic mice following betulin treatment, accompanied by an evident decrease in the proportion of Tregs within the CD4^+^ T cell population (Figure 7, C and D). Similar findings were observed in the spleen (Supplemental Figure 1, F and G). To further validate our results, we isolated Tregs from the spleens of CLP mice treated with or without betulin. Notably, these Tregs exhibited pronouncedly weakened suppressive function compared with the CLP group (Figure 7E). Furthermore, the concentrations of plasma biochemical markers associated with organ injury, including CK-MB, BUN, ALT, and AST, were markedly reduced in betulin-treated septic mice (Figure 7F). Moreover, the systemic levels of inflammatory cytokines (TNF-α, IL-6, and IL-10) were notably decreased in the botulin-treated septic mice compared with those in the control group (Figure 7G). Furthermore, histological examination of lung tissues revealed a reduction in pulmonary vascular congestion and edema in mice treated with the SREBP2 inhibitor (Figure 7H). Importantly, treatment with betulin distinctly improved the survival rate of mice with sepsis-induced multiorgan injury (Figure 7I). Collectively, our findings suggest that the inhibition of the SREBP2 pathway holds therapeutic promise in the treatment of sepsis.

## Discussion

In this study, our findings have demonstrated the pivotal role of NETs in driving the differentiation and function of Tregs, shedding light on the underlying mechanisms. Specifically, we uncovered that enhanced AKT/mTOR/SREBP2 signaling promotes cholesterol acquisition pathways in naive CD4^+^ T cells upon exposure to NETs. Furthermore, using betulin, an inhibitor of SREBP2, resulted in reduced multiple organ damage and improved survival rates in sepsis. Importantly, our findings establish a mechanistic link between NETs and the development of Tregs through cholesterol metabolism, suggesting a promising avenue for septic immunotherapy.

The innate immune response is paramount in the early stages of severe sepsis, followed by the activation of the adaptive immune system, leading to the activation of T helper cells and cytotoxic T cells (CTL) through TCR stimulation (29). Neutrophil-derived NETs have been implicated in the progression of sepsis and are known to bridge innate and adaptive immune responses by regulating CD4^+^ T cell apoptosis (29). Previous studies have demonstrated that NETs can upregulate genes involved in the regulation of Treg differentiation and function in naive CD4^+^ T cells (18). Our study observed an obvious increase in Tregs despite a decrease in total CD4^+^ T cells in late-stage sepsis, indicating that NETs may facilitate communication between the adaptive and innate immune responses by interacting with Tregs.

Cholesterol synthesis and accumulation are crucial for the proliferation and survival of activated T cells, including Tregs. Previous studies have demonstrated the crucial role of cholesterol metabolism in Treg differentiation, with distinct involvement of the mTOR signaling pathway and PPAR signaling pathway (30). Specifically, Treg proliferation requires low-level mTOR signaling, while functional activity requires heightened mTOR signaling (31). The mTORC1 complex predominantly regulates Treg growth, proliferation, and function through mevalonate/mevalonate-dependent lipid and cholesterol synthesis, exerting noticeable influence on the pathogenesis and prognosis of sepsis (32). Notably, our findings indicate that NETs serve as a metabolic switch, promoting transcription and enzymatic activity in cholesterol metabolism, thereby triggering lipid metabolic reprogramming in naive CD4^+^ T cells. The enhanced intracellular cholesterol synthesis induces Treg differentiation and suppressive activity. Mechanistically, we identified that, in sepsis, the Akt/mTOR/SREBP2 signaling pathway is augmented in naive CD4^+^ T cells, promoting cholesterol metabolism and facilitating Treg differentiation and functionality. With ongoing exploration of the lipid signaling pathways involved in Tregs during sepsis, we are getting closer to unveiling the mysteries of metabolic reprogramming. This may hold promise for the development of more effective therapeutic strategies aimed at reducing lipid signaling in Tregs, which could potentially alleviate multiorgan dysfunction in sepsis and improve patient prognosis.

Betulin, known to modulate cholesterol metabolism by targeting SREBP2, has shown efficacy in alleviating systemic inflammation and organ dysfunction in sepsis (28). In the DSS-induced colitis model, it was observed that the oral administration of betulin led to the alleviation of clinical symptoms, indicating that betulin can inhibit cholesterol biosynthesis in the intestine and ameliorate colitis (33). In the present investigation, our in vitro and in vivo data suggested that the inhibition of SREBP2 in the sepsis model suppressed systemic inflammation and mitigated vital organ dysfunction, thereby prolonging the survival of CLP mice.

TLR4, a critical immune receptor, plays a regulatory role in immune responses (34, 35). Activation of TLR4 triggers inflammatory responses and immune cell activation in the innate immune system. Additionally, we also demonstrated that TLR4 activation promotes the proliferation and stability of Tregs, enhancing their suppressive capacity. Our study also confirms that NETs influence naive CD4^+^ T cells through TLR4 signaling and inhibition of TLR4 downregulates cholesterol metabolism and reduces the proportion of differentiated Tregs. Thus, we propose that TLR4 may modulate NET-mediated immune reprogramming, bridging innate and adaptive immune responses in sepsis.

In conclusion, our findings underscore the interaction between NETs and Tregs in promoting the pathogenesis of sepsis, highlighting the upregulation of Treg cholesterol metabolism and its functional relevance. Furthermore, we underscore the potential of targeting cholesterol normalization as a therapeutic strategy for sepsis immunotherapy, and yet this hypothesis requires translational validation in the future. However, the precise mechanisms underlying the NET-mediated increase in cholesterol metabolism, leading to Treg differentiation and functional upregulation, require further exploration. Moreover, while our focus was primarily on the role of NETs in modulating Tregs in sepsis, further investigation is warranted to explore the effect of NETs on Teff regulation.

## Methods

### Sex as a biological variable.

For human participants, sex was not considered as a biological variable. Male mice were used in the experiment, because when studying immunosuppression, the immune response of male mice may be easier to control and understand because they are not influenced by the sex hormones present in female mice (36, 37). Mice (C57BL/6 background, male) were randomly assigned to all analyses.

### Human participants.

All human studies were approved by the Ethics Committee of Zhongshan Hospital, Fudan University, and performed according to the principles of the Declaration of Helsinki. This study included patients admitted to the ICU between July 2022 and June 2023 who signed a written informed consent form. Patients were diagnosed with sepsis according to the definition of sepsis 3.0, which included body temperature, heart rate, and respiratory rate (1). Exclusive criteria included history of cardiopulmonary arrest before being admitted to ICU and history of connective tissue diseases (Supplemental Table 1).

### CLP mouse model and treatments.

Ten- to twelve-week-old male C57BL/6 mice were used for the experiment. Our study was conducted in accordance with the Regulations for the Administration of Affairs Concerning Experimental Animals. After random grouping, we utilized the CLP method to induce sepsis in mice, resulting in the development of postsepsis immunosuppression by the fourteenth day according to the protocol. Briefly, after being anesthetized with 1% pentobarbital sodium (1 mg/kg), the abdominal cavity was opened. The cecum was carefully separated, ligated using 5-0 suture, and punctured with a 20-gauge needle. We then squeezed a small amount of feces and repositioned it before closing the abdominal cavity. Following the surgical procedure, each animal was administered 0.5 mL/10 g normal saline. The sham group underwent the same surgical procedure but did not undergo cecal puncture or ligation. To deplete NETs, DNase (5 mg/kg, Roche, 10104159001) was injected into the peritoneal cavity of mice. Tregs were depleted using anti-CD25 antibody (150 μg per mouse, Bioxcell, BP0012), and SREBF2 was targeted using Betulin (100 mg/kg, Sigma-Aldrich, HY14452).

### Extraction of murine bone marrow neutrophils.

After euthanizing the mice by cervical dislocation, the femurs and tibias were isolated and placed in prechilled complete 1640 culture medium (Gibco). Bone marrow was flushed out using a 1 mL needle and a 5 mL syringe, and the cell suspension was transferred to a 15 mL centrifuge tube through a 200-mesh nylon mesh filter. 80% Percoll, 65% Percoll, and 55% Percoll (Cytiva) were sequentially layered from the bottom to the top. After a 5-minute incubation, the bone marrow cell suspension was carefully added in the layer between 65% and 80% Percoll, allowing for the separation of neutrophils. The suspension was then treated with prechilled red blood cell lysis buffer to lyse erythrocytes. After 5 minutes, the cells were centrifuged and resuspended in PBS for cell counting in complete 1640 culture medium.

### Immunofluorescence.

To demonstrate the generation of NETs in response to PMA stimulation, neutrophils that had adhered to coverslips in a 24-well plate were fixed with 4% PFA, permeabilized with 0.5% Triton X-100 at room temperature for 15 minutes, and then blocked with 1% BSA at room temperature for 1 hour. Following blocking, the cells were subjected to overnight incubation at 4°C with primary antibodies targeting CitH3 (1:2,000, ab281584, Abcam) and MPO (1:100, ab90810, Abcam). After washing with PBST 3 times, Alexa Fluor 488–conjugated goat anti-rabbit IgG (1:500, GB25303, Servicebio) and Alexa Fluor Cy5–conjugated goat anti-mouse IgG (1:500, GB27301, Servicebio) were applied to the cells. DAPI was utilized for nuclear staining.

### Immunohistochemistry.

Formalin-fixed, paraffin-embedded tissue sections underwent deparaffinization and rehydration using a graded alcohol-to-water series. Subsequent to antigen retrieval and blocking procedures, the primary antibody Foxp3 (1:50, Santa Cruz, 2A11G9) and CitH3 (1:1,000, Cell Signaling Technology, 97272S) were applied to the tissue sections overnight at 4°C. Following this, an anti-rabbit HRP antibody was employed, a substrate solution was applied, nuclei were counterstained with DAPI, and the sections were dehydrated through a series of graded alcohols. The slides were visualized under a bright-field–equipped microscope.

### Naive CD4^+^ T cell isolation.

Plate coating was performed using anti-CD3 antibody (100238, Biolegend) at a concentration of 5 μg/mL, followed by overnight incubation at 4°C the day before. Mice were euthanized by cervical dislocation, and the spleens were aseptically removed. The spleens were then homogenized and filtered through a 70 μm mesh. Cell counting was performed, and the cell density was adjusted to 1 × 10^8^ cells/mL. Naive CD4^+^ T cells were isolated using the STEMCELL Naive CD4^+^ T cell Isolation Kit (19765, STEMCELL), with 5 × 10^5^ cells added per well in 500 μL culture medium containing anti-CD28 antibody (102116, Biolegend) at a concentration of 5 μg/mL. Inhibitor concentrations were maintained at 1 μM for Resatorvid (Selleck) and 1 μM for the fatostatin (MedChemExpress).

### Generation of in vitro inducible Tregs.

After naive CD4^+^ T cells were isolated using the STEMCELL Naive CD4^+^ T cell Isolation Kit (19765, STEMCELL), cells were seeded at 5 × 10^5^ per well in 500 μL culture medium containing anti-CD3/28 antibody, 0.5 ng/mL IL-2 (Biolegend, 575402), and TGF-β1 (Biolegend, 763102) at 0.5–1 ng/mL. To explore the role of NETs in iTreg differentiation, we used NETs at concentrations of 50 ng/mL, 100 ng/mL, 150 ng/mL, and 200 ng/mL DNA.

### Filipin staining.

The cells were fixed at room temperature for 30 minutes using 4% paraformaldehyde. After fixation, the cells were washed 3 times with PBS and then incubated in a solution containing 1.5 mg/mL glycine (Sangon) for 10 minutes. The cells were resuspended in PBS and incubated at room temperature in the dark with 50 μg/mL FilipinIII (B6034, APExbio) for 2 hours. Following staining, the cells were washed 3 times with PBS to remove excess dye and then they were viewed under a confocal microscope (Leica sp8, 63 × oil).

### Flow cytometry.

After processing the spleens and peripheral blood of mice into single-cell suspensions, the cells were resuspended in PBS. Red blood cells were lysed using red blood cell lysis buffer (TBD Sciences). After staining with a viability dye (423105, Biolegend), Fc block (101320, Biolegend) was employed to block nonspecific binding, and then surface antibodies were used for cell incubation. Antibodies against CD45 (103132), CD3 (100341), CD4 (100411), CD25 (102006), CD8 (100740), IFN-γ (505836), TCRγ/δ (118131), NK-1.1 (108767), CD49b (108923), CD19 (115519), CD11b (101211), IL-10 (505027), and TGF-β1 (141407) were purchased from Biolegend. Following the use of a permeabilization set (421102, Biolegend), cells were incubated with Foxp3 (320008), IL-4 (504126), IL-17 (506928), and IFN-γ (505836).

### In vitro Treg suppressive assay.

The EasySep Mouse CD4^+^CD25^+^ Regulatory T cell Isolation Kit (18783, STEMCELL) was utilized to isolate Tregs and Teffs. Teffs were resuspended in a 5 μM CFSE solution (423801, Biolegend) to achieve a concentration of 100 million cells/mL. After incubating at room temperature for 20 minutes, the reaction was terminated by adding culture medium containing 10% FBS at a volume 5 times that of the original. The cells were centrifuged at 300 g for 5 minutes, and the pellet was resuspended in prewarmed culture medium and incubated for 10 minutes. Following centrifugation, the cells were allowed to settle for 1 hour before proceeding to downstream experiments. In the NET-treated group, NETs (100 ng/mL DNA) were added to the medium for Treg culture. Tregs treated with NETs or vehicle were washed and collected for coculture with CFSE-labeled Teffs.

### Stimulation with PMA and NETs release.

Murine neutrophils were isolated as described above. After murine neutrophils were collected, they were resuspended in a 1% DMEM solution and seeded at a density of 5 × 10^5^ cells/mL in a 6-well plate. Neutrophils were stimulated with 100 nM PMA (MKbio) at 37°C for 4 hours. The upper culture medium was discarded, and cells were washed 3 times with prechilled PBS. Subsequently, 1 mL culture medium was added to each well to collect the NETs adhered to the bottom. After thoroughly pipetting down the NETs adhered to the culture dish, they were collected into a 15 mL centrifuge tube, and centrifuged at 800 g at 4°C for 5 minutes to remove cell debris. Then, we collected the supernatant for immediate use or stored it in liquid nitrogen.

### Quantification of dsDNA and MPO-DNA complexes.

The dsDNA in both human and murine plasma was quantified using the Quant-iT PicoGreen dsDNA Assay Kits and dsDNA Reagents (P7589, Thermo Fisher), following the kit’s instructions. Similarly, the concentration of MPO-DNA was determined using an ELISA kit (440007, Biolegend).

### ELISA.

Murine whole blood was centrifuged at 1,200 g for 10 minutes, and serum was collected and stored at –80°C. The levels of IL-10, IL-6, and TNF-α in mouse serum were determined using ELISA kits according to the manufacturer’s instructions (Biolegend). A total of 50 μL serum was used for one cytokine analysis. The optical density (OD) at a wavelength of 450 nm was measured.

### Western blotting.

All cells were washed with prechilled PBS and lysed using RIPA buffer (Solarbio). Protein samples were separated by 10% SDS-PAGE and transferred onto membranes. The membranes were blocked with 5% milk powder and then incubated overnight at 4°C with primary antibodies, including HMGCR (1:1,000, 13533-1-AP, Proteintech), HMGCS1 (1:1,000, 17643-1-AP, Proteintech), SREBF2 (1:1,000, 28212-1-AP, Proteintech), Phospho-PI3 Kinase p85 (1:1,000, 4228T, Cell Signaling Technology), Phospho-AKT(Ser473) (1:2,000, 66444-1-1g, Proteintech), Phospho-MTOR (Ser2448) (1:2,000, 67778-1-Ig, Proteintech), PI3 Kinase p110 (1:1,000, 27921-1-AP, Proteintech), Akt (1:5,000, 60203-2-Ig, Proteintech), Mtor (1:5,000, 66888-1-Ig, Proteintech), and β-Actin (1:3,000, 3700, Cell Signaling Technology). After washing the membranes with TBST 3 times, they were incubated with secondary antibodies at room temperature for 1 hour. The membranes were then washed and exposed using ECL. The protein bands of interest were visualized with TANON and quantitatively analyzed using Fiji.

### RNA extraction and quantitative real-time PCR.

Total RNA was extracted from cells utilizing Trizol reagent (Sigma-Aldrich), followed by purification and reverse transcription into cDNA using HiScript III All-in-one RT SuperMix, optimized for qPCR (R333-01, Vazyme), according to the manufacturer’s instructions. Subsequently, qPCR analysis was conducted employing ChamQ Universal SYBR qPCR Master Mix (Q711, Vazyme). Primers, synthesized by TSINGKE Biological Technology, are detailed in Supplemental Table 2. Relative expression levels were normalized to GAPDH for comparison of fold changes.

### Total cholesterol measurement.

Murine whole blood was centrifuged at 1,200 g for 10 minutes, and serum was collected and stored at –80°C. The concentration of total cholesterol in mouse serum was measured according to the manufacturer’s instructions (Njjcbio, A111-2-1).

### Bulk RNA-seq data.

Bulk RNA-seq data from naive CD4^+^ T cells treated with NETs or control vehicle (GEO GSE161464) were used to reanalyzed.

### Statistics.

All statistical analyses were performed using SPSS23.0 and GraphPad Prism 9 software. Our data are expressed as mean ± 95% CI. Data shown are representative of at least 3 independent experiments, and statistical significance was calculated using 2-tailed unpaired *t* test or 1-way or 2-way ANOVA. *P* values of less than 0.05 were regarded as significant.

### Study approval.

The protocols were approved by the Committee on Animal Research of Zhongshan Hospital and the Ethics Committee of Zhongshan Hospital, Fudan University.

### Data availability.

All relevant data are included in this article and its supplemental files. Values for all data points in graphs are reported in the Supporting Data Values file.

## Author contributions

Y Shi, DW, and HZ designed the experiments, offered constructive advice, and wrote the manuscript. Y Shi and DW share the co–first authorship; order of co–first authorship was determined based on their contribution to the study. Y Shi, YW, and Y Shao performed most of the experiments and analyzed the data. FZ contributed to performing the experiments. DZ, HZ, and CM offered valuable scientific discussions, supervised the study, provided scientific insight, and reviewed and edited the manuscript.

## Supplementary Material

Supplemental data

Unedited blot and gel images

Supporting data values

## Version 1 06/18/2024

In-Press Preview

## Version 2 07/22/2024

Electronic publication
