# Supplementary material for: Treg and neutrophil extracellular trap interaction contributes to the development of immunosuppression in sepsis
Source: JCI Insight. 2024 Jun 18;9(14):e180132. doi: 10.1172/jci.insight.180132 (PMC11383165; doi:10.1172/jci.insight.180132)

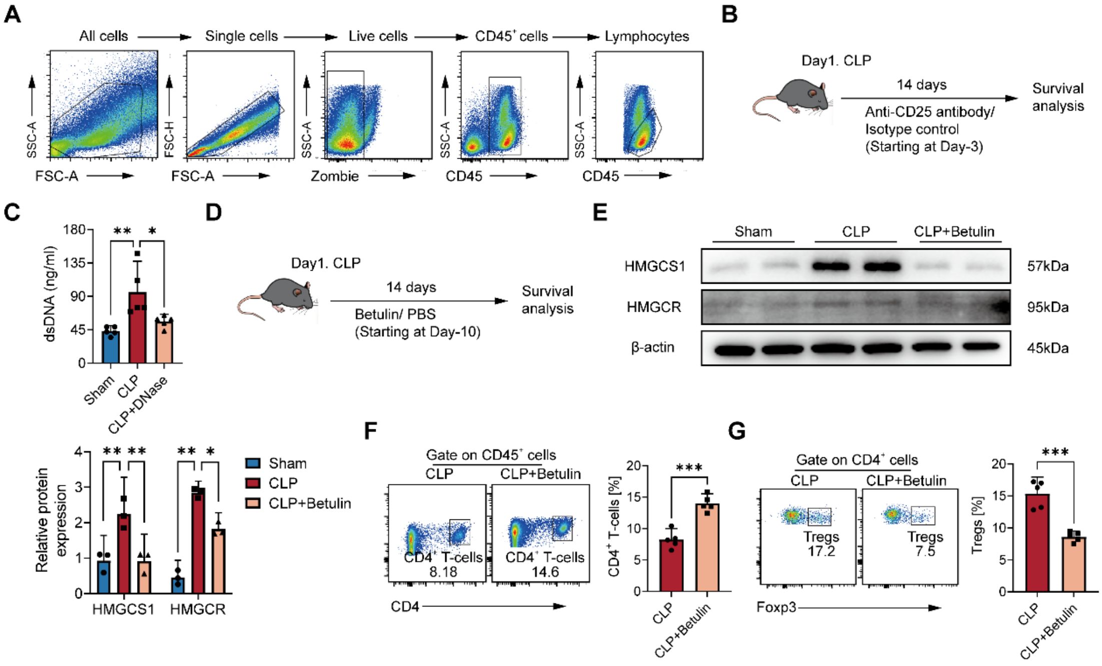

Supplementary Fig1. Analysis of lymphocytes in murine septic spleens

(A) Gating strategies of live lymphocytes in spleen and blood. (B) Immunization model of anti-CD25 antibody injection in CLP mice. (C) dsDNA levels in Sham, CLP mice treated with or without DNase (n=5). (D) Representative model of CLP mice treated with betulin. (E) Western blot images of HMGCS1 and HMGCR expression in $CD4^+$ T-cells. (F) Representative flow cytometric analysis of $CD4^+$ T-cells and (G) Treg of septic mice with betulin treatment (n=5). Each bar represents means ± 95%CI. Data comparison between two groups was analyzed by unpaired t-test. Statistical analysis for three or more groups was preformed using one-way ANOVA. $^*p < 0.05$, $^{**}p < 0.01$, $^{***}p < 0.001$, $^{****}p < 0.0001$.

Supplementary Table 1. Baseline characteristics of healthy controls (HC) and Sepsis patients.

	Healthy Control (N = 19)	ARDS (N=19)	P value
**Gender (n, %)**			0.99
**Male**	11 (57.9%)	10 (52.6%)	
**Female**	8 (42.1%)	9 (47.4%)	
**Ages (years)**	52.53±15.40	68.32±12.81	0.002
**BMI (kg/m²)**	23.72±3.47	22.66±3.24	0.335
**Neutrophils (10^9/L)**	4.65±1.11	9.64±5.76	<0.05
**Monocytes (10^9/L)**	0.41±0.15	0.51±0.44	0.315
**Lymphocytes (10^9/L)**	1.80±0.49	0.55±0.35	<0.05
**Platelets (10^9/L)**	119.53±76.61	213.95±45.99	<0.05
**Hemoglobin (g/L)**	140.63±10.78	101.74±21.30	<0.05
**Albumin (g/L)**	39.68±3.48	29.11±3.91	<0.05
**CRP (mg/L)**	2.76±3.00	55.04±24.94	<0.05

Data are expressed as n (%), mean ± SD or median (interquartile range[IQR]).

BMI: body mass index.

Supplementary Table 2. Primer sequence of q-PCR.

Gene	Forward	Reverse
**Mouse-*Srebf2***	TGGGCGATGAGCTGACTCT	CAAATCAGGGAACTCTCCCAC
**Mouse-*Hmgcr***	TGTTCACCGGCAACAACAAGA	CCGCGTTATCGTCAGGATGA
**Mouse-*Hmgcs1***	CGGATCGTGAAGACATCAACTC	CGCCCAATGCAATCATAGGAA
**Mouse-*Sqle***	GACCTCGTTCGTGACGGAC	CTCCCCAACTATCCTGTCGG
**Mouse-*Gapdh***	AGGTCGGTGTGAACGGATTTG	GGGGTCGTTGATGGCAACA

Full unedited gel for Figure 4.

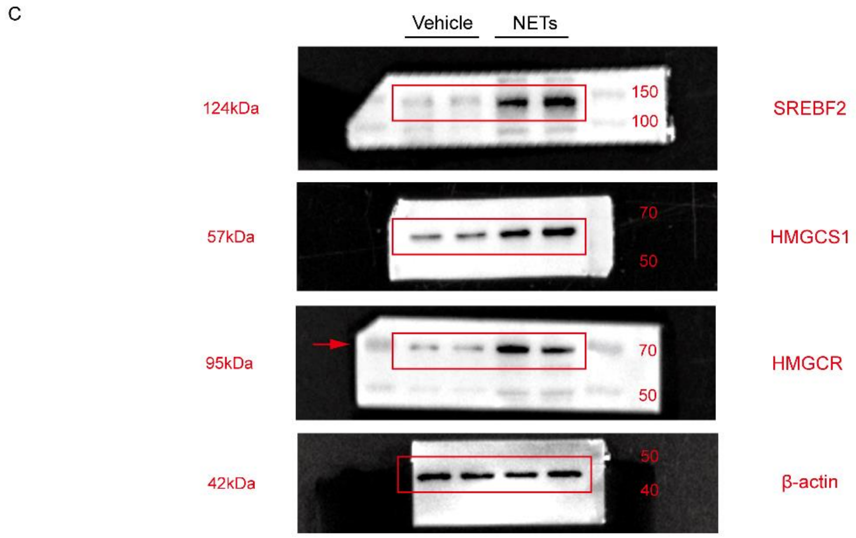

Full unedited gel for Figure 5.

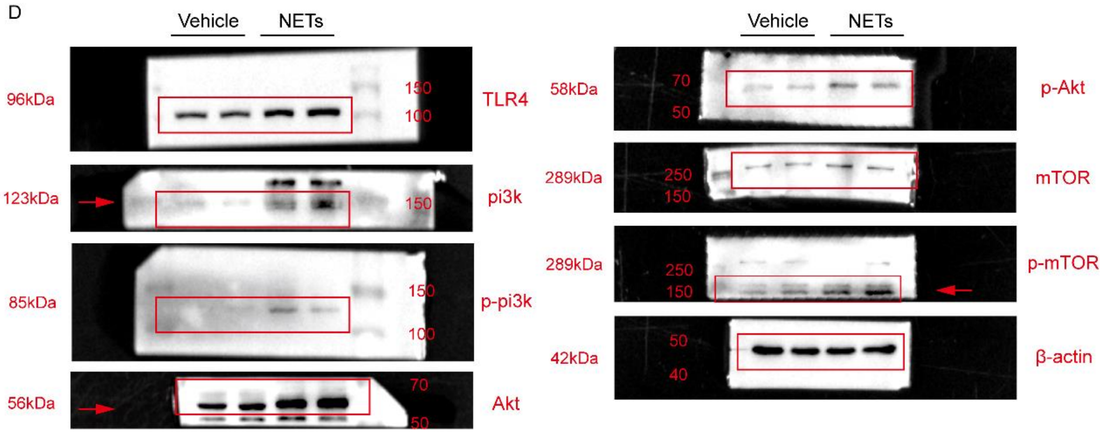

Full unedited gel for Figure 5.

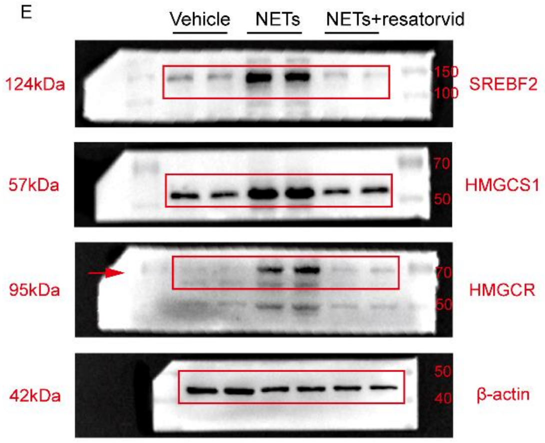

Full unedited gel for Figure 7.

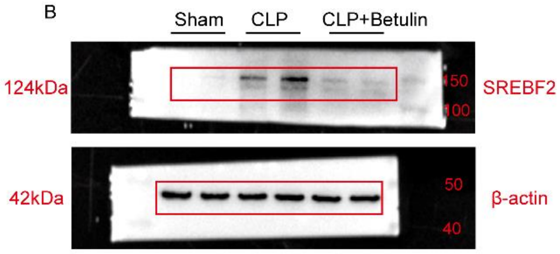

Full unedited gel for Supplement Figure S1.

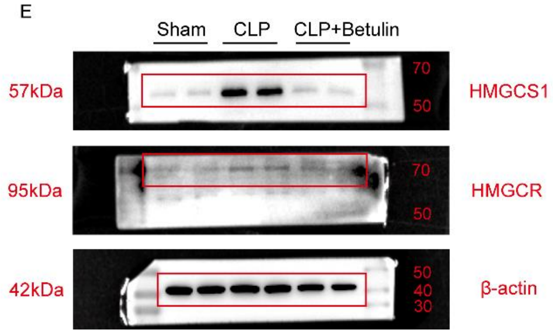

Supplement: Supplemental data [file jciinsight-9-180132-s147.pdf]
