# Supplementary material for: Treg and neutrophil extracellular trap interaction contributes to the development of immunosuppression in sepsis
Source: JCI Insight. 2024 Jun 18;9(14):e180132. doi: 10.1172/jci.insight.180132 (PMC11383165; doi:10.1172/jci.insight.180132)

Full unedited gel for Figure 4.

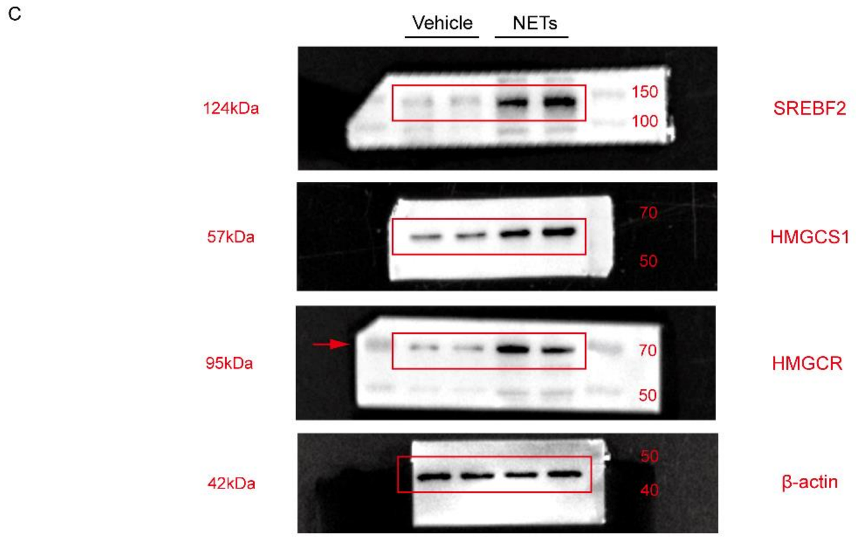

Full unedited gel for Figure 5.

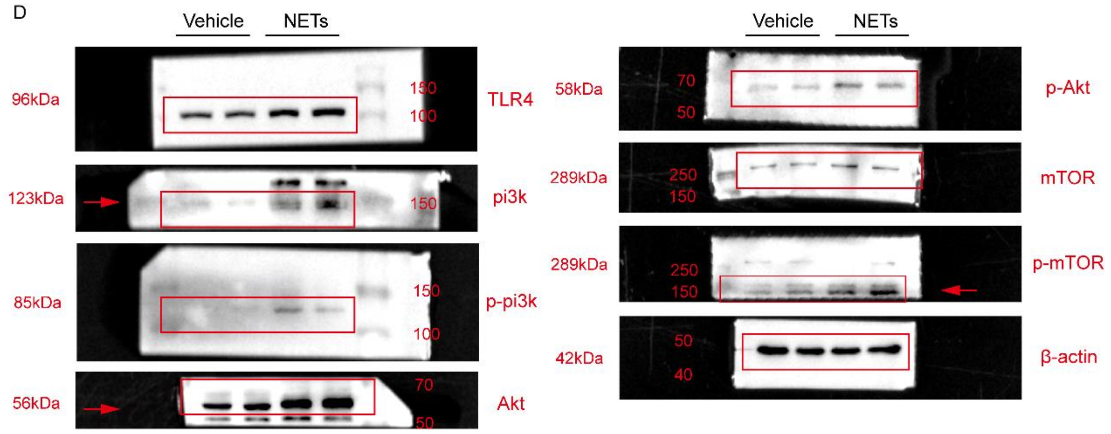

Full unedited gel for Figure 5.

E

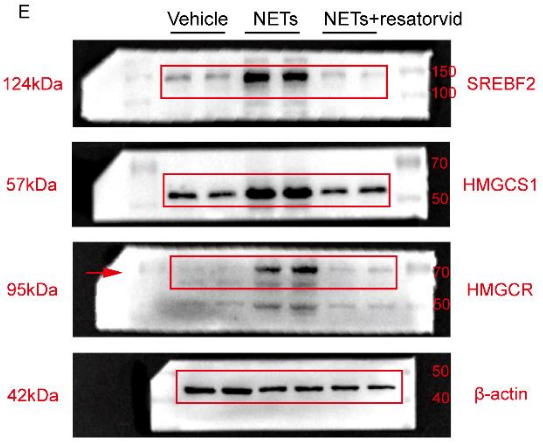

Full unedited gel for Figure 7.

B

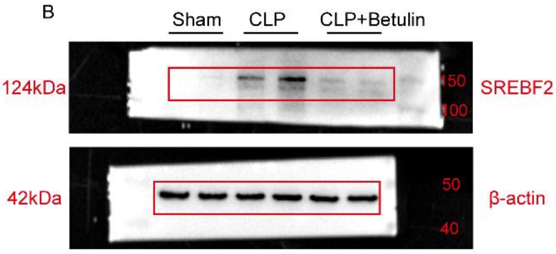

Full unedited gel for Supplement Figure S1.

E

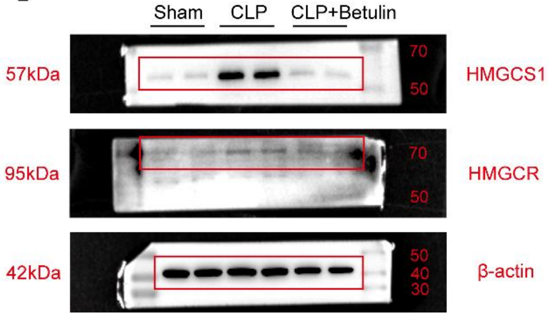

Supplement: Unedited blot and gel images [file jciinsight-9-180132-s148.pdf]
